# An eHealth Delivery Alternative for Cancer Genetic Testing for Hereditary Predisposition in Patients With Metastatic Cancers: Protocol for a Randomized Trial

**DOI:** 10.2196/72515

**Published:** 2025-08-25

**Authors:** Kimberley T Lee, Briana McLeod, Brian Egleston, Sarah Brown, Sarah Howe, Dominique Fetzer, Lauren Gutstein, Cara Cacioppo, Dana Clark, Susan M Domchek, Jessica Ebrahimzadeh, Dana Falcone, Demetrios Ofidis, Hannah Griffin, Rajia Mim, Santina Hernandez, Linda Fleisher, Kelsey Karpink, Enida Selmani, Aysha Tahsin, Lynne Wagner, Michelle Weinberg, Kuang Yi-Wen, Elisabeth Wood, Angela R Bradbury

**Affiliations:** 1 Division of Hematology/Oncology Department of Medicine University of Pennsylvania Philadelphia, PA United States; 2 Abramson Cancer Center University of Pennsylvania Philadelphia, PA United States; 3 Department of Biostatistics and Bioinformatics Fox Chase Cancer Center Philadelphia, PA United States; 4 Department of Social Sciences and Health Policy Division of Public Health Sciences Wake Forest School of Medicine Winston Salem, NC United States; 5 Department of Medical Oncology Thomas Jefferson University Hospital Philadelphia, PA United States; 6 Department of Medical Ethics and Health Policy University of Pennsylvania Philadelphia, PA United States

**Keywords:** genetic testing, genetic counseling, digital health, metastatic cancer, digital genetic education

## Abstract

**Background:**

Germline *BRCA1* and *BRCA2* testing is a standard evidence-based practice, with established risk reduction and cancer screening guidelines for genetic carriers. With Food and Drug Administration approval for poly (adenosine diphosphate ribose) polymerase (PARP) inhibitors in patients with metastatic breast, ovarian, pancreatic, and prostate cancer, there is an additional therapeutic rationale for testing all patients with these cancers for germline *BRCA1* and *BRCA2* mutations. However, many at-risk patients do not have access to genetic services, leaving many genetic carriers unidentified.

**Objective:**

The eREACH (A Randomized Study of an eHealth Delivery Alternative for Cancer Genetic Testing for Hereditary Predisposition in Metastatic Breast, Ovarian, Prostate, and Pancreatic Cancer Patients) study evaluates the effectiveness of a theoretically and stakeholder-informed eHealth (eg, digital) delivery alternative to traditional genetic counseling for patients with metastatic breast or prostate cancer or advanced or metastatic ovarian or pancreatic cancer referred for genetic testing to determine whether they are candidates for a PARP inhibitor.

**Methods:**

The eREACH study is a randomized noninferiority study using a 2 × 2 design to test a self-directed digital intervention to deliver clinical genetic testing for patients with metastatic cancers. The traditional standard-of-care pretest (visit 1) and posttest (visit 2—disclosure) counseling delivered by a genetic counselor is replaced with our patient-informed digital intervention. The four arms were as follows: arm A, genetic counselor for visits 1 and 2; arm B, genetic counselor for visit 1 and digital intervention for visit 2; arm C, digital intervention for visit 1 and genetic counselor for visit 2; and arm D, digital intervention for both visits. Participants were adults with advanced or metastatic breast, ovarian, pancreatic, and prostate cancer. The primary outcomes of this study were change in genetic knowledge and anxiety from baseline to postdisclosure assessment. We will test whether the digital intervention is noninferior to standard-of-care counseling with a genetic counselor using a modified noninferiority ANOVA of the posttest disclosure minus baseline change scores. In secondary analyses, we will test pairwise differences among the 4 groups.

**Results:**

As of January 2025, we have completed enrollment of 229 participants. Data analysis is ongoing, and we expect the results to be published in 2025.

**Conclusions:**

Increasing indications for *BRCA1* and *BRCA2* testing create a pressing need to evaluate alternative delivery models to increase access and uptake of these tests while maintaining adequate patient cognitive, affective, and behavioral outcomes. The eREACH study evaluates the effectiveness of an interactive, patient-centered digital intervention to deliver clinical genetic testing to patients with metastatic cancers. We expect that this work will inform evidence-based guidelines and the standard of care for delivery of genetic testing, and it is designed to be broadly applicable and easily adaptable for other populations and settings even beyond oncology.

**Trial Registration:**

ClinicalTrials.gov NCT04353973; https://clinicaltrials.gov/study/NCT04353973

**International Registered Report Identifier (IRRID):**

DERR1-10.2196/72515

## Introduction

### Metastatic Cancer and Genetic Testing

Increasing indications for *BRCA1* and *BRCA2* testing creates a pressing need to evaluate alternative delivery models for testing. Germline cancer genetic testing has become a standard evidence-based practice, with established risk reduction and cancer screening guidelines for genetic carriers [1-4]. With Food and Drug Administration approval for poly (adenosine diphosphate ribose) polymerase (PARP) inhibitors as treatment for patients with germline *BRCA1* and *BRCA2* mutations and metastatic breast, ovarian, pancreatic, and prostate cancer, there is an additional therapeutic rationale for testing patients with these cancers for germline *BRCA1* and *BRCA2* mutations [5-8]. However, many at-risk patients do not have access to genetic services, leaving many genetic carriers unidentified [9-13].

### Traditional Model of Genetic Services

The traditional standard of care for genetic counseling services involves a patient having a pretest counseling session with a certified genetic counselor (GC). Genetic testing is then performed, following which there is a second visit with a certified GC where results are shared or disclosed. Thus, one test has traditionally required 2 visits with a GC. While this is the traditional model for services, many alternative models, including mainstreaming (oncologist provides all counseling) or replacing pretest counseling with videos, have been recently used, although many with limited comparison to the traditional model [14-21]. Furthermore, with the 21st Century Cures Act, results may become available in the medical record before the traditional disclosure with a GC. Access to genetic specialists is limited in many areas in the United States, and the traditional delivery model of pre- and posttest counseling with a genetic professional will not support the rising indications for *BRCA1* and *BRCA2* testing. With current policy movement toward testing all patients with breast, ovarian, pancreatic, and prostate cancer for germline mutations driven by advances in treatment with PARP inhibitors or other targeted therapies, there is a need to develop and evaluate innovative delivery models for genetic testing for *BRCA1* and *BRCA2* and other targeted mutations. This could become even more important if guidelines are modified to include additional tumor types for which PARP inhibitors could be effective based on *BRCA1* and *BRCA2* status [22].

### eHealth-Based Genetic Services

eHealth (eg, digital) interventions are a novel and efficient delivery model with potential to increase access and throughput of genetic services. One alternative, efficient, and scalable model is to provide predisclosure education and return of genetic results through secure eHealth (eg, digital) interventions. Interactive health communication applications (eg, digital applications) have been implemented to support and enhance the delivery of patient-centered care and communication to optimize health outcomes [23,24]. Patients have high interest in communicating electronically with health care providers; 85% of patients report preferring electronic communication to phone [25-27]. Studies in a variety of disease settings have shown that digital interventions can improve knowledge, social support, self-efficacy, and some clinical outcomes [23-35]. A 2023 systematic review by Lee et al [36] identified 70 digital tools, of which 32 were designed to help with decision-making regarding genetic testing. Of these, only 11 were developed for posttest counseling, and approximately half were used in an oncology setting. However, these tools all had a neutral or favorable effect on knowledge and decision-making. Recently, Biesecker et al [16] reported that a web-based platform was noninferior to GC-mediated genetic counseling in a highly educated cohort undergoing carrier testing, and the Returning Genetic Research Panel Results for Breast Cancer Susceptibility (RESPECT) study [37] demonstrated that a web-based predisclosure education intervention in lieu of standard predisclosure counseling with a GC was acceptable to participants, with 88.5% opting for web disclosure. Thus, interactive digital interventions may be an efficient way to address the workforce shortage and other challenges in the delivery of genetic services while maintaining adequate patient outcomes [38-40].

### Conceptual Model

Our patient-reported outcomes of interest in the eREACH (A Randomized Study of an eHealth Delivery Alternative for Cancer Genetic Testing for Hereditary Predisposition in Metastatic Breast, Ovarian, Prostate, and Pancreatic Cancer Patients) study were informed by our conceptual model grounded in the self-regulation theory of health behavior to evaluate innovations in the delivery of genetic services [16,41-44]. This model proposes that an individual’s understanding, knowledge, and perception of the disease threat and risk reduction behaviors (cognitive outcomes) drives the reaction to (affective outcomes) and use of health (genetic) information (behavioral outcomes) [42-44]. It focuses on lay representations instead of medical and scientific definitions. It further addresses the potential for individual variability in understanding by incorporating individual-level cognitive, emotional, familial, and cultural experiences. Thus, it has been proposed that the self-regulation theory of health behavior is an ideal framework for considering the outcomes of genetic testing when testing alternative delivery models [42,45-47].

### This Study

This randomized study of an eHealth (eg, digital) delivery alternative for cancer genetic testing for hereditary predisposition in patients with metastatic breast, ovarian, prostate, and pancreatic cancers (eREACH) is a 4-arm noninferiority study with a 2 × 2 design in a population of patients with metastatic cancers to evaluate the effectiveness of digital delivery alternatives to increase access to genetic testing. Participants will be randomized to 1 of 4 arms. The control group will receive both pretest and posttest genetic counseling from a certified GC per the traditional standard of care. The intervention arms will receive pretest or posttest counseling via our interactive, patient-centered digital intervention.

### Objectives

#### Overview

The goal of the eREACH study is to evaluate the effectiveness of replacing traditional pretest (visit 1—education and consent) and posttest (visit 2—disclosure) counseling delivered by a GC with an interactive patient-centered digital intervention for germline genetic testing in patients with advanced or metastatic cancers for which a PARP inhibitor could be considered for treatment. We hypothesize that offering digital alternatives for pretest or posttest (result disclosure) visits will result in equal or improved (noninferior) short-term and longitudinal cognitive, affective, and behavioral outcomes and reduce health care provider time, increasing throughput and access to genetic services. Furthermore, we will evaluate subgroups who benefit more or less from digital alternatives, which will provide critical information on how best to implement and increase access to germline genetic testing in the era of precision medicine.

#### Specific Aim 1

Our primary aim is to determine whether a patient-centered digital intervention delivery of pretest or posttest counseling can yield equal or improved short-term cognitive and affective outcomes as compared to a 2-visit telehealth delivery model with a GC. Our primary outcomes are change in knowledge and general anxiety from baseline to postdisclosure assessment. Secondary outcomes include uptake of testing, depression, cancer-specific distress, uncertainty, change in treatment plan, communication of results, and health care provider time.

#### Specific Aim 2

Our secondary aim is to evaluate patient and delivery factors that moderate the impact of digital delivery alternatives on short-term and longitudinal cognitive, affective, and behavioral outcomes and health care provider time.

## Methods

We used the SPIRIT (Standard Protocol Items: Recommendations for Interventional Trials) checklist when writing this report [48].

### Study Design

**Figure 1:**
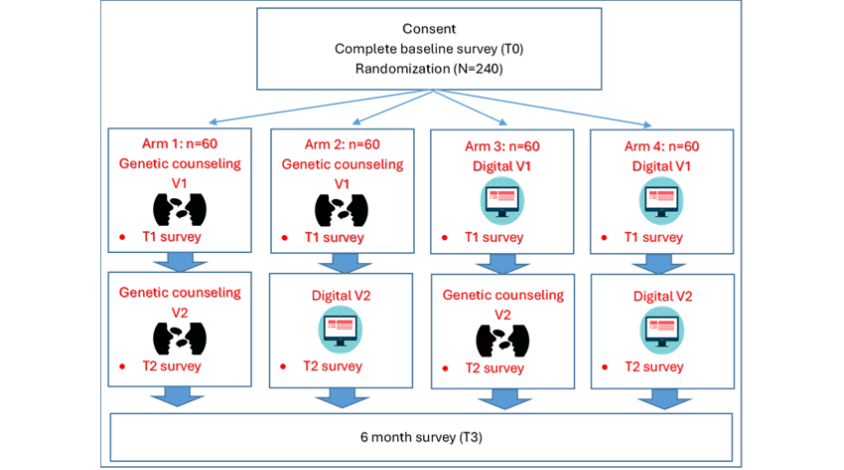
Study schema. Genetic counseling was available for all eHealth (digital) visits upon request. V1: visit 1 (pretest education); V2: visit 2 (result disclosure).

eREACH is a noninferiority study using a 2 × 2 design where traditional standard-of-care pretest (visit 1—education and consent) or posttest (visit 2—disclosure) counseling delivered by a GC are replaced with a patient-centered digital intervention (Figure 1). All patients randomized to the digital intervention can request to speak with a GC instead of completing the digital intervention or at any time during the process.

### Ethical Considerations

This study has been approved by the University of Pennsylvania Institutional Review Board (protocol 833370). Any major protocol modifications will be reported in subsequent manuscripts describing study results. Recruitment emails to eligible participants include the study purpose and procedures as well as the direct REDCap (Research Electronic Data Capture; Vanderbilt University) web link to the electronic informed consent form, Health Insurance Portability and Accountability Act (HIPAA) document, and clinical consent to care (for patients new to Penn Medicine). Recruitment letter recipients can (1) immediately consent to the eREACH study directly using the provided electronic link, (2) request to speak with the research team for additional information, or (3) decline participation. Research staff follow up via email and telephone with individuals who do not respond. Individuals are given the option of signing a paper consent document if preferred. Reasons for declining are recorded, and all decliners are reminded that they can still receive counseling and testing services through usual resources. In addition, given the potential urgency for testing in patients with metastatic or advanced cancer, a verbal consent option is offered to interested participants not able to complete the electronic informed consent form and HIPAA document to reduce barriers to participation and testing. To ensure confidentiality, all responses and case characteristics will be deidentified. Participants will receive a US $10 gift card for each survey completed. With 4 surveys available, participants could receive up to US $40 in total for taking part in this study.

### Patient-Centered eHealth Intervention

**Figure 2:**
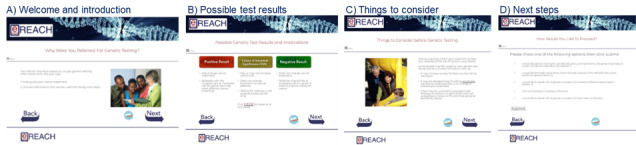
Examples of screens from the pretest counseling (visit 1) digital intervention.

The patient-centered digital intervention is informed by the tiered-binned model and designed to replace a traditional counseling session for patients who find this to be sufficient information. The intervention includes tier 1 (indispensable information presented to all users) and optional tier 2 content (more in-depth information, examples, and videos). Completing tier 1 takes 11.5 to 14 minutes for the average user, and it takes 28 minutes to review all content and videos. Readability testing was completed for all content, and modifications were made to achieve a readability score of eighth grade or lower when possible. As shown in Table 1, the pretest intervention includes eight modules: (1) introduction, (2) purpose of testing, (3) genetics overview, (4) implications of results, (5) possible results and implications, (6) genetic testing options, (7) things to consider before genetic testing, and (8) testing decision. Example screenshots are shown in Figure 2.

After viewing the minimum tier 1 screens, participants are asked to record their decision to either proceed with testing and their testing choice (targeted test only, gene panel testing, or large or custom panel testing) *or* decline testing and their reason. Those who log a decision to move forward with testing have their selection, along with the details of their personal and family history, reviewed by a study GC. In the event that a study GC feels that an alternative decision for testing may be more appropriate based on family history, patients will receive a call and speak with the GC to confirm their decision.

**Figure 3:**
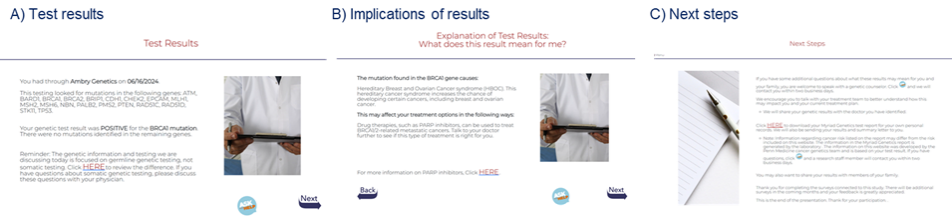
Examples of screens from the post-test counseling (visit 2) digital intervention.

The patient-centered digital result disclosure (visit 2) intervention includes four modules: (1) welcome page and instructions, (2) test result, (3) explanation of result, and (4) next steps (Table 2). Completing tier 1 once takes approximately 4 to 5 minutes for the average user, and it takes approximately 9 to 10 minutes to review all content and videos. Readability testing was completed for all content, and modifications were made to achieve a readability score of eighth grade or lower when possible. Example screenshots are shown in Figure 3.

**Table 1:** eREACH (A Randomized Study of an eHealth Delivery Alternative for Cancer Genetic Testing for Hereditary Predisposition in Metastatic Breast, Ovarian, Prostate, and Pancreatic Cancer Patients) patient-centered eHealth pretest education (visit 1) intervention.

	Tier 1 content^a^	Tier 2 content	Tier 2 videos
Module 1—introduction	Landing page and welcome (1 screen) What is the eREACH study? (1 screen) How to use this website (1 screen) What to expect (overview of content and option to log testing decision at the end; 1 screen)	—^b^	—
Module 2—purpose of testing	Role of genetic testing for patients with metastatic cancer (1 screen) How testing might affect cancer treatment (1 screen)	More information about PARPc inhibitors (1 screen) More information about immunotherapy (1 screen)	—
Module 3—genetics overview	Genes and mutations (4 brief sliding screens) Difference between tumor (eg, somatic) and inherited genetic changes (1 screen) How inherited mutations are passed down through families (1 screen) Difference between sporadic and inherited cancers and examples and BRCA1 and BRCA2 example (2 screens) Types of inherited cancer risk genes (high risk, moderate risk, and limited information; 1 screen)	More information on inherited gene risks (4 screens), including high-risk genes (1 screen), moderate-risk genes (1 screen), and limited-information risk genes (1 screen) Risks of cancer with each of the aforementioned genes (1)	Basics of genetics (1 min, 19 s)
Module 4—implications of results	How their results might impact their medical care (1 screen) How their results might impact their family’s medical care (1 screen)	More information about PARP inhibitors (1 screen) More information about immunotherapy (1 screen) More information on cancer risks by gene and risk reduction options for relatives (1 screen)	—
Module 5—possible results and implications	Types of results (positive, negative, and VUSd) and what they mean (1 screen)	What is a VUS? (1 screen)	What is a VUS? (3 min, 47 s)
Module 6—genetic testing options	3 testing options: targeted, panel, or customized panel (1 screen) Benefits and limitations of each option (1 screen) Choosing the test that is right for them (1 screen) Things to consider before genetic testing (1 screen)	How do patients decide on testing options (3 screens), including choosing option 1 (focused on treatment now; 1 screen), choosing option 2 (multigene panel; 1 screen), and choosing option 3 (customizing with a GCe)	Genetic test panel options (2 min, 36 s) Choosing a custom panel (1 min, 51 s)
Module 7—things to consider before genetic testing	Risks and limitations of testing (2 screens) Costs (billed to insurance; patients will be contacted if costs are of >US $100; 1 screen)	What is a VUS? (1 screen) Types of uncertainty (3 screens), including VUS (1 screen); uncertainties about risks or best medical management (1 screen); and uncertainty because family history does not match the gene mutation, with CDH1 example (1 screen) More information on insurance coverage, financial assistance, and option to speak with a GC about potential costs (1 screen)	What is a VUS? (3 min, 47 s)
Module 8—testing decision	Next steps (GC review, sample collection, and average time for results; 1 screen) Whether the patient has enough information and testing choice (2 screens) Testing choice (1 screen)	—	—
Optional content	—	Glossary of terms	—

^a^Completing tier 1 content takes 11.5-14 minutes to complete for the average user, and 28 minutes for all content and videos. Readability testing was completed for all content and modifications made to achieve a readability score of 8^th^ grade or lower when possible.

^b^Not applicable.

^c^PARP: poly (adenosine diphosphate ribose) polymerase.

^d^VUS: variant of uncertain significance.

^e^GC: genetic counselor.

**Table 2:** eREACH (A Randomized Study of an eHealth Delivery Alternative for Cancer Genetic Testing for Hereditary Predisposition in Metastatic Breast, Ovarian, Prostate, and Pancreatic Cancer Patients) patient-centered eHealth result disclosure (visit 2) intervention.

	Tier 1 content^a^	Tier 2 content	Tier 2 videos
Module 1—welcome page and instructions	Overview and navigation instructions, including how to ask for help (1 screen)	Reason for genetic testing (1 screen) More information about PARPb inhibitors (1 screen) More information about immunotherapy (1 screen) Genetics overview (1 screen) Types of results (positive, negative, and VUSc) and what they mean (1 screen) What is a VUS?	Genetic counselor explaining “a brief genetics lesson” (1 min, 19 s) What is a VUS? (3 min, 47 s)
Module 2—test result	Genetic test result and summary of genes tested (1 screen)	Difference between tumor genetic changes (eg, somatic) and inherited genetic changes (1 screen)	—^d^
Module 3—explanation of result	“What does this result mean for me?” (2 screens) Follow-up recommendations (1 screen) “What does this mean for my relatives?” (1 screen), including specific cancer risks for relatives if the patient tests positive (1 screen) and medical recommendations (1 screen)	For positive results: what is a PARP inhibitor (1 screen) and more about cancer risk management for relatives (1 screen) For VUS results: what is a VUS? (1 screen)	What is a VUS? (3 min, 47 s)
Module 4—next steps	Informing the patient that results have been shared with their health care provider and to talk with them about implications for treatment; recommendation to share the results with family members (1 screen)	PDF of clinical test result available for download	—

^a^Completing Tier 1 content once takes approximately 4-5 minutes for the average user, and approximately 9-10 minutes for all content and videos. Readability testing was completed for all content and modifications made to achieve a readability score of 8^th^ grade or lower when possible.

^b^PARP: poly (adenosine diphosphate ribose) polymerase.

^c^VUS: variant of uncertain significance.

^d^Not applicable.

### Design and Development of the Web Intervention

The initial intervention content was reviewed with patients with cancer purposively selected to represent a range of ages, sexes, educational levels, and cancer types through individual user testing interviews (Table 3). We included both participants who had previously had and not had genetic counseling and testing to ensure a diverse range of perspectives in their feedback. Participants were asked to comment on the clarity, usefulness, and appearance of each screen. They were also asked to comment on proposed video topics, including usefulness, what they would expect to see covered in the video, and the likelihood of them watching the video. A total of 11 participants reviewed the pretesting education (visit 1) intervention content, and 8 reviewed the result disclosure (visit 2) intervention content. Interviews were reviewed to identify recommendations for changes or areas of confusion. Two research team members reviewed and selected key comments for consideration, and at least 2 research team members and a GC reviewed comments to consider changes to the content. All specific recommendations were considered, particularly cases in which more than one participant recommended a similar change. In scenarios in which user testing participants had conflicting recommendations, the team used best judgment to make changes. The most common recommendations to both the visit 1 and visit 2 interventions were to simplify content (eg, reduce words or details), address confusing content, change the order of the content, or move content to tier 2, as well as increase representativeness in photos, examples, and content.

**Table 3:** Characteristics of the participants who completed the eREACH (A Randomized Study of an eHealth Delivery Alternative for Cancer Genetic Testing for Hereditary Predisposition in Metastatic Breast, Ovarian, Prostate, and Pancreatic Cancer Patients) user and usability testing.

Characteristic		User testing^a^ (n=19)	Usability testing^b^ (n=18)
Age (y), range		31-72	31-83
**Sex, n (%)**			
	Female	13 (68)	8 (44)
	Male	6 (32)	10 (56)
**Race, n (%)**			
	Non-White	1 (5)	0 (0)
	White	17 (89)	18 (100)
	Declined to answer	1 (5)	0 (0)
**Educational level, n (%)**			
	Some college or lower	3 (16)	2 (11)
	College degree	4 (21)	5 (28)
	Advanced degree	12 (63)	11 (61)
**Personal history of cancer, n (%)**			
	Metastatic breast cancer	5 (26)	4 (22)
	Advanced or metastatic ovarian cancer	3 (16)	1 (6)
	Metastatic prostate cancer	5 (26)	7 (39)
	Advanced or metastatic pancreatic cancer	6 (32)	6 (33)
**Previous testing, n (%)**			
	Yes	13 (68)	15 (83)
	No	3 (16)	3 (17)
	In process^c^	3 (16)	0 (0)

^a^In total, 11 participants reviewed visit 1 content, and 8 participants reviewed visit 2 content.

^b^Nine participants reviewed visit 1 content, and 9 participants reviewed visit 2 content.

^c^Had had pretest counseling or blood drawn but no disclosure.

We completed usability testing with 18 participants, 9 (50%) who completed the visit 1 intervention and 9 (50%) who completed the visit 2 intervention (Table 3). These participants used the digital intervention and were asked to comment on things that were confusing, functions that were challenging, and other changes they suggested. A research assistant viewed their navigation through cobrowsing software. Several function challenges were identified, prompting revisions to the intervention. These included addressing clearer instructions and labeling for arrows, hyperlinks, and hover functions and addressing how to return to the main content after accessing tier 2 content. Some minor changes to content addressed participant comments regarding typos, confusing terms, and increasing representation in images and content. Representative user comments that resulted in key changes to the intervention are described in Table 4.

**Table 4:** Summary of key changes made during development and design of the web intervention based on user comments during user and usability testing.

Key changes recommended		Representative user comments
**User testing**		
	Simplify content	“Don’t like the small print. With ‘chemobrain’ this would be frustrating. Make it simple.”
	Address confusing content	“...where it says ‘unaffected family members have/will not have the option to pursue genetic testing.’ According to what? Needs more clarification.”
	Change order of the content	“Mention the glossary earlier on in the ‘how to use this website’ page so people know it is there from the get-go.”
	Move content to tier 2	“Would like it to be shorter—what do people really need? Maybe make tiered 1 info as short as possible (10 slides) and everything else tier 2.”
**Usability testing**		
	Clearer instructions and labeling	“Thought it would be nice to have text accompanying it [arrows] like the words ‘next’ and ‘previous.’”
	Hyperlinks and hover functions	“Would like cursor to change when hovered over the menu—to indicate that you could click on it.” “On iphone—can’t read instruction to hover over—too small.”
	Returning to main content from tier 2	Some confusion about tier 2 loops—sometimes the forward arrow gets you back to where you started. Sometimes there is only a back arrow.”
	Correction of typos and confusing terms	“‘Targeted therapy’ is not a phrase most people understand.” “Would expect more expanation or link to glossary for ‘microsatilite instability.’”
	Increase representativeness in photos	“Noted throughout the images were of older folks and predominately white. Felt it should be more diverse to normalize genetic testing for a wider audience.”

### Setting

Remote genetic counseling services in this study are provided through the Penn Telegenetics Program and Penn Cancer Risk Evaluation Program. The Penn Telegenetics Program has developed procedures for individual patients and health care providers across the nation to access remote genetic counseling services. For patients outside Pennsylvania, the Penn Telegenetics GCs collaborate with local health care providers to provide genetic services in the home. This model includes a physician registration process to facilitate this collaborative care model and has been successfully used in related nationally recruiting studies (NCT04455698 and NCT05427240). The Penn Telegenetics GCs are licensed in all US states as required by state licensure laws [40].

### Study Participants

#### Eligibility

Eligibility criteria include individuals diagnosed with metastatic breast or prostate cancer or advanced or metastatic ovarian or pancreatic cancer who are aged ≥18 years, able to understand and communicate in English, and residing in the United States. Exclusion criteria include uncorrected or uncompensated speech defects that would lead to the participant being unable to communicate effectively with a medical provider; uncontrolled psychiatric or mental conditions; or severe physical, neurological, or cognitive deficits rendering the individual unable to understand study goals or tasks. Participants who have already received cancer germline genetic testing are excluded.

#### Recruitment

Eligible patients can be referred by a health care provider or self-referred from a range of recruitment sources. First, at the University of Pennsylvania and affiliate oncology programs, members of the research team review treating physicians’ schedules to identify potentially eligible patients and, with health care provider permission, contact them for enrollment. Health care providers in these programs can also directly refer patients to the research team. Second, the Penn Telegenetics Program provides remote clinical services at several regional centers without GCs on-site. Clinical coordinators and health care providers at these sites identify eligible patients in their practices and refer them to the research team. Third, cancer-specific support groups (eg, Facing Our Risk of Cancer Empowered and Cancer Support Community) advertise the eREACH study through study flyers and website banners. Patients can contact the eREACH team and enter the study as self-referrals. Finally, in collaboration with the University of Pennsylvania Office of Clinical Research, the team posts Facebook advertisements on ClinicalTrials@Penn. Interested patients can enter the study as self-referrals.

Health care providers who refer eligible patients can complete an online permission-to-contact registration form to provide contact information for the patient. Research staff then contact the patient via phone or email, which includes the study purpose and procedures and a REDCap link to the electronic informed consent form and HIPAA document. Potential participants receive up to 5 contacts, typically 3 emails and 2 phone calls. Recruitment invitations began in September 2020.

#### Enrollment Goals

As outlined in the following sections, we initially planned enrollment of 560 participants (120 participants per arm). Given the low initial recruitment (2020-2023), which was attributed to the COVID-19 pandemic, and an increase in mainstream testing in oncology practices (oncology health care providers ordering genetic testing without GCs), we re-evaluated our initial enrollment goals (see the Data Analysis Plan section). In December 2023, we adjusted our enrollment goal to 60 participants per arm with baseline (T0) and postdisclosure visit 2 (T2) data (at least 240 in total).

### Study Arms

#### Overview

Once participants have completed informed consent, the baseline (T0) survey, and family history collection with research staff, they are randomized into 1 of the 4 study arms. Randomization is stratified by sex and cancer type using a permuted block design within strata for randomization. The allocation sequence was generated by the study biostatistician and made available to research staff.

#### Pretest Counseling (Visit 1)

Participants in the genetic counseling arms (A and B) complete their pretest counseling session via phone or videoconference (according to patient preference) in their home or their selected location. Penn Telegenetics GCs use communication protocols adapted from related studies [18,43]. The Penn Telegenetics team contacts participants up to 5 times to schedule their pretest counseling session. If a scheduled participant does not show up for their appointment, they are contacted 3 times to reschedule. If visit 1 is not completed, the corresponding health care provider is notified (if the participant was health care provider referred). Participants who select videoconference are provided links to download secure videoconferencing software on their home computer or device. The Penn Telegenetics team uses a HIPAA-compliant technology platform for videoconference visits. In our communication protocols, if videoconference technology fails, GCs convert the session to phone.

Participants randomized to the digital intervention arms (C and D) are provided with instructions for obtaining a private code to access the pretest counseling digital intervention. Participants are informed that they can choose to schedule a counseling session with a GC if they do not wish to access the digital intervention or do not have online access. They can also ask to speak with a GC at any point.

Participants are asked to complete a post–visit 1 survey (T1) after completing counseling or the visit 1 digital intervention. While participants will be asked to complete the survey within 72 hours (3 days), surveys that are completed up to 7 days after consent are still accepted.

#### Genetic Testing

**Figure 4:**
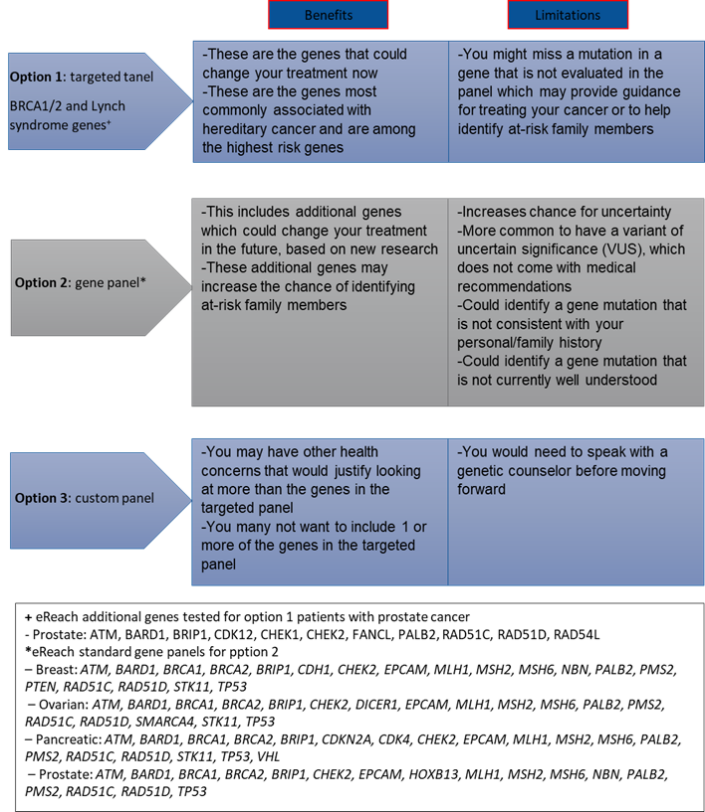
Participant options for genetic testing. eREACH: A Randomized Study of an eHealth Delivery Alternative for Cancer Genetic Testing for Hereditary Predisposition in Metastatic Breast, Ovarian, Prostate, and Pancreatic Cancer Patients.

In all study arms, there are 3 available options for testing (Figure 4). Option 1 is a targeted panel limited to genes that could immediately impact treatment for metastatic cancer (*BRCA1* and *BRCA2* and Lynch syndrome). Additional homologous recombination repair genes will be tested for patients with prostate cancer based on the Food and Drug Administration label for the use of PARP inhibitors in this tumor type. Option 2 is a larger gene panel including additional genes related to their cancer type that could have an impact on treatment in the future or additional impact for relatives. Option 3 is a customized panel based on other health concerns or a desire to exclude particular genes. For option 3, participants are asked to speak with a GC to review their concerns and options. Those who log a decision on the digital intervention (arms C and D) have their selection, along with the details of their personal and family history, reviewed by their assigned study GC. In the event that the GC feels that an alternative decision for testing may be more appropriate (ie, the participant selects the targeted panel when a large or custom panel may be indicated), the GC calls the participant to confirm their decision. Self-referred participants are asked to provide contact information for an oncologist (or other health care provider) to serve as the ordering health care provider with the GC as required by state licensure laws. The Penn Telegenetics clinical team contacts the health care provider’s office to give them information about the program and procedures and confirm the health care provider’s information to include in the testing order.

Consistent with current clinical care, all genetic testing will be sent to a commercial laboratory (eg, Invitae or Ambry Genetics) and covered by insurance. At the time, laboratories would notify patients if out-of-pocket expenses of >US $100 were expected. Patients are instructed to contact the GC for any questions regarding coverage and cost. Test kits are sent to the participants’ home, and participants receive as many as 3 reminders to return their test kits. When requested, patients can have their samples collected at the University of Pennsylvania phlebotomy laboratory.

#### Disclosure of Results (Visit 2)

Those randomized to genetic counseling for visit 2 (arms A and C) are contacted via phone by their assigned GC to share their results. These are not scheduled calls, and GCs make up to 5 attempts over 10 days (routine cases) and up to 3 attempts over 3 days (urgent cases). For the disclosure sessions, a visual aid packet that is publicly available for clinical use, is provided to all participants, GCs complete disclosure checklists, and all sessions are recorded unless participants decline.

Those randomized to the digital intervention for visit 2 (arms B and D) are provided with their result and gene-specific screens (as appropriate), which are first reviewed by 2 study team members (a research team member and the GC) for accuracy and quality control. Results are uploaded into the digital intervention by a study team member. Participants are notified via email that results are available and can be accessed and that they can request to speak with a GC if they prefer before or after viewing their results. After return of results, the GC provides the referring or registered health care provider with the genetic test results and chart note. GCs are available to answer any patient or health care provider questions. Study team members contact patients via email or phone up to 5 times every 2 to 3 days for those who have not accessed the digital intervention after their results have been released. If results are not viewed after these reminders, the participants are contacted by their assigned GC to attempt to disclose results. If this is unsuccessful, a notification is sent to the treating physician to make them aware, and the results are provided to the treating physician. As of September 2023, all results have been also uploaded to the electronic medical record in accordance with Penn Medicine’s policies to comply with the 21st Century Cures Act.

### Outcomes

#### Uptake of Testing and Patient-Reported Outcomes (Aim 1)

Theoretically informed patient-reported cognitive, affective, and behavioral outcomes of genetic services will be collected at baseline (T0), after visit 1 (T1) and visit 2 (T2), and at 6 months (T3).

Uptake of counseling and testing is assessed through Penn Telegenetics service records for those assigned to (or who select) standard-of-care genetic counseling. Uptake for those assigned to the digital intervention will be recorded as completion of the digital intervention.

Understanding of genetic information is assessed at all time points (T0-T3). Knowledge of genetic disease will be evaluated using The KnowGene Scale, a 16-item scale administered to patients after genetic counseling and testing to measure their understanding of the health implications of genetic test results. It includes health implications to oneself as well as relatives and covers penetrance, actionability, limitations of current technology, and monogenic inheritance patterns [49].

Reactions to genetic information are assessed using multiple instruments that evaluate psychological distress, adjustment, and satisfaction:

- General anxiety and depression are assessed using the 4-item short Patient-Reported Outcomes Measurement Information System [50,51].
- Disease-specific distress is measured using the 8-item Impact of Event Scale [52-55], which has strong internal consistency (Cronbach α=0.82-0.90) in genetic delivery studies [56-58].
- Satisfaction with genetic services is assessed using 14 items evaluating satisfaction with genetic services (T1 and T2). These items have been used in our related studies evaluating alternative and traditional genetic testing delivery models (Cronbach α=0.73-0.85) and adapted for digital delivery [17,56,57,59-62].
- Multidimensional responses to genetic testing, including positive responses and uncertainty, will be assessed using the Multidimensional Impact of Cancer Risk Assessment questionnaire at T2 and T3. The Multidimensional Impact of Cancer Risk Assessment questionnaire has been used in many genetic studies to evaluate distress, uncertainty, and positive responses to receipt of genetic test results [63]. Four items were excluded as they were not relevant to patients with metastatic cancer.
- Decisional regret (T2 and T3 only) is assessed using the 5-item validated Decision Regret Scale used frequently in related genetic studies [64,65].

Use of genomic information includes change in treatment plan and communication of results (T3) to health care providers, family members, and others using items adapted from our related studies. For all actionable results (*BRCA1* and *BRCA2* and Lynch syndrome genes), we either confirm changes in the treatment plan in the medical record (Penn Medicine patient and health care provider participants) or by contacting the treating oncologist for confirmation of whether the patient received a PARP inhibitor or immunotherapy and, if not, why.

Health care provider time is recorded per patient for a subset of participants to estimate health care provider time by arm. This includes time for remote telehealth counseling. For patients in arms B to D, where services have the potential to be completed partially or entirely by the digital intervention, these patients will still have a GC review their history, decision, and support testing submission; review results and the disclosure information provided; and be available for questions upon request. Thus, in all arms, GC time for case review, genetic counseling, and conversations with patients and their health care providers will be recorded for a subset of patients.

#### Moderators of Uptake and Patient-Reported Outcomes (Aim 2)

Moderators are collected at baseline and include the following:

- Sociodemographic data, including race and ethnicity, educational level, marital status, sex, and age.
- Health literacy—assessed using 3 Brief Health Literacy Screen items that have been validated to detect inadequate health literacy in clinical medical populations [66].
- Comfort with technology—assessed using 8 selected Health Information National Trends Survey items [67].
- Psychological impacts due to the COVID-19 pandemic—assessed in a subset of patients (start of recruitment until May 2023) using the Social Psychological Measurements of COVID-19: Coronavirus Perceived Threat, Government response, Impacts, and Experiences Questionnaires, a 14-item measure previously published by the University of Montana in April 2020. While this protocol is independent of COVID-19, it was advised to use a measure that may help assess for psychological distress that is due to the COVID-19 pandemic separate or in addition to any psychological impacts from receiving genetic testing results [68].

In addition, individual use of the digital intervention is recorded throughout the study to evaluate the impact of intervention use (eg, dosage) on outcomes and who benefits more and less from the intervention. Intervention use includes actual use, adherence, and attrition [69,70]. For actual use, we record the time when each participant logs in and logs out. Actual use will be evaluated for the intervention in general and secondarily for specific intervention content (eg, videos and specific pages). Adherence will be defined as completing all of tier 1 at least once.

### Data Analysis Plan

#### Primary Noninferiority Analysis

We hypothesize that the intervention arms incorporating the digital delivery intervention, with access to a GC upon request, can yield equal or improved short-term and longitudinal cognitive, affective, and behavioral outcomes and lower health care provider time. Our primary outcomes are change between T0 and T2 in knowledge and anxiety. We will test whether the digital intervention is noninferior to standard-of-care counseling with a GC. In noninferiority tests, the null and alternative hypotheses are the reverse of the usual; in this case, the null hypothesis is that the web intervention is worse than the standard of care. We will conclude that the web intervention is noninferior using a modified noninferiority ANOVA of the T2 minus T0 change scores. More details on the power and type I error are provided in the following section. We will use an intention-to-treat approach comparing randomization arms, but we will also confirm results in secondary per-protocol analyses; many recommend that both intention-to-treat and per-protocol analyses be consistent to declare noninferiority [71]. In secondary analyses, we will test pairwise differences among the 4 groups. For longitudinal analyses across the 3 survey times (T0-T3), we will examine time trajectories using regressions estimated through generalized estimating equations to account for within-subject correlation over time. We will include main and interaction terms of arm with time panel indicators.

Our noninferiority test will be based on an ANOVA *F* statistic test jointly comparing the 4 randomization arms, in which we declare noninferiority if the joint *P* value is of >.15 and all 3 experimental web arms have an observed standardized effect of −0.25 SD units or better (with the direction standardized such that higher values imply more beneficial change) when compared to the control standard of care–only arm. If the ANOVA *F* test *P* value is ≤.15, then we will declare noninferiority if the 3 standardized one-sided 85% confidence bounds for the 3 intervention arms relative to the controls are >−0.35 SDs. We define standardization as rescaling variables to have an SD of 1 relative to the pooled baseline SD (see more details in the following section).

#### Sample Size Justification for Aim 1

We originally anticipated 140 participants per arm (560 in total). We chose our 2 primary variables as they demonstrated possible differences between groups in preliminary data from a prior study in which genetic test results were returned to patients; in this study, we expect that harmful changes will not occur. We will use a noninferiority test and, to be conservative, will require noninferiority in both the intention-to-treat and per-protocol analyses, as recommended [71].

With 140 people per arm, we have <1% noninferiority type I error and >82% noninferiority power if we simply use the first criteria that the *P* value must be >.15 and all 3 intervention arm coefficients must be >−0.25 (ie, in the direction away from being harmful when compared to the standard-of-care group). If we further apply the decision rule that the 85% one-sided CIs are bounded away from −0.35 if *P*≤.15, the power increases to 94%, and the type I error rate remains at <1%.

In calculating the type I error rate, we assumed that the 4–change score standardized means (standardized to have an SD of 1) for the null hypothesis were 0 (arm A=control group), 0.3 (single web intervention arms), and −0.6 (double web intervention arm), with negative values indicating a harmful effect. Our alternative hypothesis was a null effect (4–change score means of 0). These were conservative estimates of changes based on standardized RESPECT web intervention findings of a change score movement of 0.3 in anxiety and a change score movement of 0.4 in knowledge. Hence, we have excellent noninferiority power. Calculations were made using PASS (version 11; NCSS Statistical Software) in conjunction with R simulations (R Foundation for Statistical Computing). In secondary analyses, we will examine pairwise comparisons among the 4 arms using 2-tailed *t* tests.

In Multimedia Appendix 1, we provide the probability of declaring noninferiority under a range of assumptions about the intervention effects relative to the standard-of-care control arm. We see that our probability of declaring noninferiority is appropriate under several assumptions about population-level mean differences among arms.

On the basis of low recruitment, we conducted an unplanned conditional power calculation (December 1, 2023) using data recorded until October 24, 2023, to determine whether it would be appropriate to end recruitment at 60 participants per arm. The statistician and the principal investigator were unblinded to the analysis, but the rest of the staff and coinvestigators were blinded. We used the same decision rules for declaring noninferiority as mentioned previously. We present the findings in Table 5. In the “Original assumptions” section, we replicated the findings of the original power calculations (Multimedia Appendix 1) but, instead, assumed a sample size of 60 participants per arm. In the “Genetic knowledge” and “PROMIS anxiety” sections, we conditioned on the probability of declaring the study noninferior on the observed data from the first participants enrolled through October 2023. Next, we simulated data for the future participants based on the assumptions in each row. We added the simulated data to the observed data and tested our noninferiority hypothesis. We iterated the simulations 50,000 times, creating simulated future participant data each time, and calculated the proportion of iterations in which we would conclude noninferiority. Conditioning on the data obtained until October 24, 2023, we have excellent power under assumptions of no effect or beneficial effects or an assumption that the future data will have the same average responses as the data collected until October 24, 2023. For anxiety, the noninferiority type I error rate (ie, the probability of declaring the study noninferior if the intervention is harmful) is still relatively low, which is appropriate.

Regarding standardization, we will standardize the change scores with respect to the baseline SD among the 4 groups (ie, before patients have received any component of the intervention). Hence, our power calculations assume change score SDs of 1, which would be the case if the within-person correlation of measurement between periods is 0.5 (SD of change score = 1 + 1 − 2 × 0.5 × 1 = 1). If the correlation is of >0.5, as may likely be the case, the power will be better.

Regarding the choice of −0.25 as a lower noninferiority bound, we chose −0.25 as an absolute 0.25 standardized effect is often considered a small effect. Hence, our noninferiority margin only tolerates small negative effects. If the *P* value is of ≤.15, we nonetheless ensure that the bound of the one-sided CI is >–0.35, which is still of modest magnitude.

Regarding the choice of our alternative hypothesis, it assumes that all average change scores are 0 (4 mean changes of 0). This is conservative as it does not assume any benefit of the interventions when compared to arm A (the control group). If the web intervention does have a beneficial effect, the power for the noninferiority design will be even better, as shown in Multimedia Appendix 1 and Table 5.

**Table 5:** Probability of declaring the study noninferior under various assumptions about standardized change score means (N=60 per arm; SD 1 for all mean values).

General assumption		Arm A (control), mean (SD)	Arm B (web visit 2), mean (SD)	Arm C (web visit 1), mean (SD)	Arm D (web visits 1 and 2), mean (SD)	Probability of declaring the study noninferior (%)
**Original assumptions (unconditional on observed data)**						
	No effect (power)	0	0	0	0	79
	Arm B and arm C beneficial	0	0.2	0.2	0	86
	Web intervention slightly beneficial	0	0.1	0.1	0.1	92
	Heterogeneous effects	0	−0.1	0.1	0	71
	Web intervention slightly harmful	0	−0.1	−0.1	−0.1	59
	Web intervention harmful—small effect	0	−0.2	−0.2	−0.2	36
	Web intervention harmful—relatively small effect	0	−0.25	−0.25	−0.25	25
	Web intervention harmful—modest effect	0	−0.3	−0.3	−0.3	16
	Only Arm B harmful	0	−0.35	0	0	23
	Web intervention harmful—moderate effect	0	−0.35	−0.35	−0.35	9
	Arm D very harmful (type I error)	0	−0.3	−0.3	−0.6	2
**Genetic knowledge** **—** **conditional on observed data (higher scores are better)**						
	No effect	0	0	0	0	99
	Arm B and arm C beneficial	0	0.2	0.2	0	100
	Web intervention slightly beneficial	0	0.1	0.1	0.1	100
	Heterogeneous effects	0	−0.1	0.1	0	99
	Web intervention slightly harmful	0	−0.1	−0.1	−0.1	99
	Web intervention harmful—small effect	0	−0.2	−0.2	−0.2	97
	Web intervention harmful—relatively small effect	0	−0.25	−0.25	−0.25	96
	Web intervention harmful—modest effect	0	−0.3	−0.3	−0.3	95
	Only arm B harmful	0	−0.35	0	0	86
	Web intervention harmful—moderate effect	0	−0.35	−0.35	−0.35	93
	Arm D very harmful	0	−0.3	−0.3	−0.6	97
	Observed effects until October 2023	0	Blinded	Blinded	Blinded	98
**PROMIS** ^a^ **anxiety (conditional on observed data; direction of effect reversed so that higher scores are better)**						
	No effect	0	0	0	0	92
	Arm B and arm C beneficial	0	0.2	0.2	0	91
	Web intervention slightly beneficial	0	0.1	0.1	0.1	96
	Heterogeneous effects	0	−0.1	0.1	0	89
	Web intervention slightly harmful	0	−0.1	−0.1	−0.1	84
	Web intervention harmful—small effect	0	−0.2	−0.2	−0.2	72
	Web intervention harmful—relatively small effect	0	−0.25	−0.25	−0.25	64
	Web intervention harmful—modest effect	0	−0.3	−0.3	−0.3	56
	Only arm B harmful	0	−0.35	0	0	73
	Web intervention harmful—moderate effect	0	−0.35	−0.35	−0.35	47
	Arm D very harmful	0	−0.3	−0.3	−0.6	17
	Observed effects until October 2023	0	Blinded	Blinded	Blinded	98

^a^PROMIS: Patient-Reported Outcomes Measurement Information System.

#### Missing Data

We will conduct complete case analyses for the primary analyses. If there are substantial missing data, we will repeat analyses using the multiple imputation methods by Raghunathan et al [72]. The multiple imputation analyses will be considered secondary sensitivity analyses to investigate how missing data might be affecting inferences.

#### Additional Secondary Analyses for Aim 1 (Amendment Added in July 2023)

As treatment effects might differ with changes in procedures related to the 21st Century Cures Act period (before vs after the implementation of immediate genetic test result release to patients at the University of Pennsylvania), we will estimate interaction regression models of the primary and secondary end point in which we include dichotomized period (0 or 1; no or yes; before July 2023 vs after July 2023), randomization arm, and the interaction between period and randomization arm (randomization arm indicators × period) as covariates in the model. In addition, we will examine interaction models in which we compare outcomes of people who receive results before their study protocol disclosure method appointment with those who do not receive results before their study protocol disclosure method appointment. We will exclude those who do not receive results at all in this secondary analysis examining the potential moderation effect of preappointment disclosure.

#### Moderator Analysis

We will evaluate moderators of outcomes, identifying those who benefit more and less from the intervention in clinical practice. For moderation (ie, effect modifier analyses), we will include in the generalized estimating equations–estimated regressions indicators (0 or 1 variables) denoting randomization arms, the potential moderator variable, and interaction terms between the moderators and randomization indicators (ie, an interaction term will be created by multiplying 2 terms). We will further examine 3-way interaction models in which we include time interactions. To maintain power to detect moderation, we will examine nontime moderators separately. We will also examine adding potential confounders to models. For all analyses, we will examine effects after assigning participants to intention-to-treat, per-protocol, and as-treated groups.

## Results

IRB approval was obtained in June 2019 and recruitment began in August 2020. We have completed the enrollment of 256 study participants and data collection was completed in September 2024. Data analysis is ongoing and we expect results to be published in Fall 2025.

## Discussion

### Expected Findings

We hypothesize that our digital delivery of pretest or posttest genetic counseling will yield equivalent or improved outcomes compared to the traditional 2-visit standard-of-care delivery model with a GC. This model has the potential to expand access to genetic services by leveraging digital technologies. For patients with metastatic cancers, access to genetic services is a necessary step to gain access to targeted therapeutic treatments, and our model may lead to improved access to targeted therapy reliant on germline genetic testing. We hypothesize that participants randomized to digital genetic counseling arms will experience noninferior changes in genetic knowledge and general anxiety compared to those randomized to the standard 2-visit genetic counseling model. We also predict that uptake of genetic testing, depression, cancer-specific distress, and health care provider time will be equivalent or improved in digital arms compared to the standard-of-care arm. Furthermore, we will explore demographic and clinical factors associated with uptake of the intervention and testing.

Several related studies looking at various alternative delivery models were ongoing at the time the eREACH study was designed and started recruiting [19-21,36,71,73]. Two studies among patients with prostate cancer (Technology-Enhanced Acceleration of Germline Evaluation for Therapy [TARGET] and ProGen) have been completed and found that digital pretest educational tools were noninferior to traditional genetic counseling [19,20]. TARGET used an educational web tool, and ProGen tested an educational video that was delivered to participants in person. Posttesting disclosure was performed by a GC in both studies [19,20]. The Making Genetic Testing Accessible (MAGENTA) study demonstrated that omitting pretest counseling for all participants and posttest counseling for participants without a pathogenic variant was noninferior to phone-based genetic counseling in terms of posttest distress [21]. MAGENTA included patients with breast cancer (3.3% of the total cohort) or a family history suggestive of a hereditary cancer syndrome but not patients with advanced or metastatic cancer. In addition, participants were highly educated (88.9% with some college education or higher) and mostly White individuals (95.3%) and likely represent an early adoption population; findings may be different in a diverse clinical population of patients with advanced cancer. While these studies support the premise that digital delivery alternatives may play a role in clinical genetics, there are no published randomized studies comparing the outcomes of using digital alternatives for returning genetic results to those of the traditional model of genetic counseling in metastatic cancer genetics (where testing is more complex and routinely now includes at least an option for multigene panels) and in diverse patient populations more reflective of real-world clinical practice. The eREACH study will address this gap in the literature by evaluating the efficacy of the proposed patient-centered digital delivery of pretest or posttest counseling for genetic testing among patients with metastatic breast or prostate cancer or advanced or metastatic ovarian or pancreatic cancer.

Another frequently endorsed model to increase uptake of testing is “mainstreaming,” wherein the traditional pretesting counseling session with a GC is replaced with educational materials and a conversation with a cancer care provider, such as an oncologist [15,74]. This provider orders the genetic test that they deem to be most appropriate, and results are disclosed by the medical provider with the option for a GC on patient request, when a positive result or a variant of uncertain significance is identified, or in the setting of a family history of cancer or a known familial mutation. A recent systematic review and meta-analysis identified 29 studies on mainstreaming, of which only 5 assessed patient-reported outcomes such as anxiety, depression, and decisional conflict [74]. Importantly, there are no published studies comparing mainstreaming to the traditional 2-visit genetic counseling model in which multigene panel genetic testing is offered. However, some of these studies include models that can be compared to arm C of the eREACH study (web intervention and GC), and we will be able to compare our patient-reported outcomes to those of studies that reported similar longitudinal measures.

The findings of this study will help determine whether the use of digital delivery of genetic counseling is an effective model for patients with metastatic cancers. If deemed noninferior, the eREACH study will provide an evidence base for a scalable digital model for genetic counseling for diverse and real-world patients with cancer in academic and community settings.

### Strengths and Limitations

We may encounter potential challenges in this study. Loss to attrition for the surveys may result in missing data. We will monitor completion rates during our quality control checks and assess and address barriers to survey completion during the study period. Inability to contact treating health care providers could result in missing data regarding the use of genetic test results for participants outside of the Penn Medicine system. Some health care providers may not be willing to serve as the ordering health care provider for genetic testing services. Although we have not experienced significant challenges with local physicians being willing to register with the Penn Telegenetics program, identifying and understanding barriers to oncologist uptake would be important for future implementation of this model. The financial burden of genetic testing may limit patient access to these services. We do not anticipate that this will be a common issue, but understanding these barriers to testing would also be important for future iterations of our delivery model. The inclusion of self-referred participants has the potential to introduce a selection bias wherein patients with higher digital literacy may be more likely to participate in this study. Given that this is a randomized trial, such participants should be evenly distributed between study arms, thus only potentially posing a threat to generalizability. To address generalizability, we will compare the digital literacy of the study population to that of the general population using available normative data from the Health Information National Trends Survey. Finally, our delivery model includes the involvement of a GC (even if there is no direct contact with the patient), whereas mainstream testing, with no involvement of GC, has become increasingly common. While we will not be able to conduct direct comparisons to these different delivery models in this randomized trial, we expect to be able to compare our results to those of other studies that may evaluate the outcomes of mainstream testing, assuming that they assess similar patient-reported outcomes and use similar measures.

### Future Directions

The eREACH study will provide critical empirical data on the effectiveness of an interactive patient-centered digital intervention to deliver clinical genetic testing to patients with metastatic cancers. We expect that this work will inform evidence-based guidelines and the standard of care for delivery of genetic testing, and it is designed to be broadly applicable and easily adaptable for other populations and settings even beyond oncology.

## Appendix

Multimedia Appendix 1 Original power calculations: probability of declaring noninferior under various assumptions about standardized change score means. N=140/arm.

## Abbreviations

eREACH: A Randomized Study of an eHealth Delivery Alternative for Cancer Genetic Testing for Hereditary Predisposition in Metastatic Breast, Ovarian, Prostate, and Pancreatic Cancer Patients
GC: genetic counselor
HIPAA: Health Insurance Portability and Accountability Act
MAGENTA: Making Genetic Testing Accessible
PARP: poly (adenosine diphosphate ribose) polymerase
REDCap: Research Electronic Data Capture
RESPECT: Returning Genetic Research Panel Results for Breast Cancer Susceptibility
SPIRIT: Standard Protocol Items: Recommendations for Interventional Trials
TARGET: Technology-Enhanced Acceleration of Germline Evaluation for Therapy

## Data Availability

The datasets generated or analyzed during this study are not publicly available as participants were not consented with knowledge of data sharing, but summary deidentified data are available from the corresponding author on reasonable request. The study principal investigator will have access to the final trial dataset.
