# Supplementary material for: An eHealth Delivery Alternative for Cancer Genetic Testing for Hereditary Predisposition in Patients With Metastatic Cancers: Protocol for a Randomized Trial
Source: JMIR Res Protoc. 2025 Aug 25;14:e72515. doi: 10.2196/72515 (PMC12417906; doi:10.2196/72515)
Supplement: Multimedia Appendix 1 [file resprot_v14i1e72515_app1.docx]

| **General Assumption** | **Mean**  **Arm A (Control)** | **Mean**  **Arm B**  **(V2 Web)** | **Mean**  **Arm C**  **(V1 Web)** | **Mean**  **Arm D**  **(V1+V2 Web)** | **Probability of declaring non-inferior** |
| --- | --- | --- | --- | --- | --- |
| No effect (power) | 0 | 0 | 0 | 0 | 94% |
| Single web beneficial | 0 | .2 | .2 | 0 | 97% |
| Web slightly beneficial | 0 | .1 | .1 | .1 | 99% |
| Heterogeneous effects | 0 | -.1 | .1 | 0 | 85% |
| Web slightly harmful | 0 | -.1 | -.1 | -.1 | 75% |
| Web harmful (small effect) | 0 | -.2 | -.2 | -.2 | 40% |
| Web harmful (small effect) | 0 | -.25 | -.25 | -.25 | 23% |
| Web harmful (moderate effect) | 0 | -.3 | -.3 | -.3 | 11% |
| Single arm harmful | 0 | -.35 | 0 | 0 | 15% |
| Web harmful (moderate effect) | 0 | -.35 | -.35 | -.35 | 5% |
| Arm 4 very harmful (Type I error) | 0 | -.3 | -.3 | -.6 | <1% |
